# The Influence of the Val158Met Catechol-O-Methyltransferase Polymorphism on the Personality Traits of Bipolar Patients

**DOI:** 10.1371/journal.pone.0062900

**Published:** 2013-04-30

**Authors:** Wendy Dávila, Nieves Basterreche, Aurora Arrue, María I. Zamalloa, Estíbaliz Gordo, Ricardo Dávila, Miguel A. González-Torres, Mercedes Zumárraga

**Affiliations:** 1 AMSA, Bilbao, Spain; 2 Unidad de Hospitalización de Corta Estancia, Red de Salud Mental de Bizkaia, Hospital de Zamudio, Zamudio, Bizkaia, Spain; 3 Departamento de Psiquiatría, Universidad del País Vasco, Leioa, Bizkaia, Spain; 4 Departamento de Investigación Neuroquímica, Red de Salud Mental de Bizkaia, Hospital de Zamudio, Zamudio, Bizkaia, Spain; 5 Departamento de Psiquiatría, Hospital de Basurto, Bilbao, Spain; The Nathan Kline Institute, United States of America

## Abstract

**Introduction:**

Certain personality traits and genetic polymorphisms are contributing factors to bipolar disorder and its symptomatology, and in turn, this syndrome influences personality. The aim of the present study is to compare the personality traits of euthymic bipolar patients with healthy controls and to investigate the effect of the catechol-O-methyltransferase (COMT) Val158Met genotype on those traits. We recruited thirty seven bipolar I patients in euthymic state following a manic episode and thirty healthy controls and evaluated their personality by means of the Cloninger’s Temperament and Character Inventory (version TCI-R-140). We assessed the influence of the polymorphism Val158Met in the COMT gene on the personality of these patients. The patients scored higher than controls in harm avoidance (61.3±12.5 vs. 55.3±8.1) and self-transcendence (45.3±12.8 vs. 32.7±8.2) and scored lower than controls in self-directedness (68.8±13.3 vs. 79.3±8.1), cooperativeness (77.1±9.1 vs. 83.9±6.5) and persistence (60.4±15.1 vs. 67.1±8.9). The novelty seeking dimension associates with the Val158Met COMT genotype; patients with the low catabolic activity genotype, Met/Met, show a higher score than those with the high catabolic activity genotype, Val/Val.

**Conclusions:**

Suffering from bipolar disorder could have an impact on personality. A greater value in harm avoidance may be a genetic marker for a vulnerability to the development of a psychiatric disorder, but not bipolar disorder particularly, while a low value in persistence may characterize affective disorders or a subgroup of bipolar patients. The association between novelty seeking scores and COMT genotype may be linked with the role dopamine plays in the brain’s reward circuits.

## Introduction

Bipolar disorder (BD) is a complex syndrome with different subgroups, episodes and symptoms that contribute to its varied clinical features and course. Evidence from family, twin, and adoption studies suggests that BD has a strong genetic component; however, specific genes that contribute to the illness remain unclear, and the etiopathogenesis of the disorder is still unknown. Several studies have attempted to identify biological substrates that could provide an objective basis for the diagnosis and classification of BD. One strategy involves the study of personality traits.

It has been proposed that personality may predispose a person to BD [1]. It is also possible that personality affects the clinical features of BD and/or that BD symptomatology influences personality. Initial studies concluded that the personality traits of euthymic bipolar patients and normal controls were similar; however, differences in personality have been found even in mood-stabilized patients [2], [3], [4], [5]. When the patients are not euthymic [6], it is difficult to dissect the effect of personality from symptomatology [7].

Cloninger and colleagues proposed a biosocial model of personality. According to this model, temperament and character dimensions interact to form an individual’s personality. It includes four independent heritable dimensions of temperament: novelty seeking, harm avoidance, reward dependence and persistence. Cloninger and colleagues hypothesized positive correlations between serotonergic activity and harm avoidance, dopaminergic activity and novelty seeking, and noradrenergic activity and reward dependence, however the evidence supporting these assumptions is not definitive. The character traits self-directedness, cooperativeness, and self-transcendence are considered, according the model, to be the result of both genetic and environmental influences.

BD with psychotic features has been suggested to be a subtype of BD [8] with specific personality characteristics; in the prodromal phase, increased energy/goal–directed activity was found more frequently in patients with subsequent psychotic BD than in patients with non-psychotic BD [9]. Psychotic symptoms have been associated with brain dopaminergic activity [10]. Subcortical dopaminergic supersensitivity consequent of a cortical hypodopaminergic state has been hypothesized to underlay the appearance of psychotic symptomatology. In the prefrontal cortex, dopamine is mainly inactivated extraneuronally via the catechol-O-methyltransferase (COMT) enzyme; dopamine transporter is scarce, and thus, dopamine reuptake is almost nonexistent. Within the COMT gene, the rs4680 single nucleotide polymorphism influences the enzyme’s activity. This polymorphism consists of a replacement of guanine with adenosine, resulting in a substitution of the amino acid valine (Val) for methionine (Met). The COMT enzyme is more active in Val/Val homozygotes than in Met/Met homozygotes, and heterozygotes display intermediate levels of COMT enzymatic activity [11]. This polymorphism has been associated with a patient susceptibility to developing psychotic symptoms [12], [13] and certain personality traits [14].

The aim of the present study is to compare the personality traits in euthymic BD patients and healthy controls as well as to investigate the effect of the COMT Val158Met genotype on those traits.

## Materials and Methods

### 1. Patients and Controls

Ethics Statement: This study was approved by the ethics committee of the Hospital of Galdakao and conducted according to the Declaration of Helsinki. Patients were duly informed before giving their written consent.

We selected patients of Western European descent admitted into the acute psychiatric unit of the Hospital of Zamudio, Bizkaia, Spain diagnosed with bipolar I disorder, manic episode, according to the DSM-IV-TR [15] using the Structured Clinical Interview [16]. After receiving pharmacological treatment with olanzapine and lithium, the patients were considered to be euthymic when they scored <10 in the Young Mania Rating Scale [17] and <8 in the Hamilton Scale for Depression [18]. Once euthymia was established, the personality traits were evaluated in 37 patients through the Cloninger’s Temperament and Character Inventory (version TCI-R-140) [19].

We selected 30 controls of Western European origin. The control group received no pharmacological treatment and had no personal or family history of psychiatric disorders. The personality traits were evaluated through the TCI-R-140.

### 2. Evaluation of COMT Gene Polymorphism

A heparinized blood sample was obtained from all patients between 8 and 8.30 am. DNA was purified from the blood using a commercial kit (Nucleospin Blood, Macherey and Nagel, Düren, Germany). The COMT genotype was assessed by real-time PCR using a commercial kit (Life Technologies, Carlsbad, CA, USA).

### 3. Statistical Analysis

Statistical analysis of the data was performed using the Statgraphics Plus computer program (Statpoint Technologies, Warrenton, VA, USA). The level of significance was set at p<0.05. The TCI scores of the patients and controls were compared using Student’s t test or the Mann-Whitney test. We constructed a general linear model in which the dependent variables were the seven items of the TCI and the factors included the Val158Met COMT genotype and gender; age was included as a co-variable. A post hoc analysis was performed using the Bonferroni method. Gender and age were included in the analysis because they may influence some personality traits [20], [21].

## Results

The description of the group of patients and controls is presented in Table 1. There were no significant differences in gender distribution (χ^2^ = 0.04, p = 0.84) or mean age (t = 0.24, p = 0.80) between the patients and controls. Thirty patients had suffered psychotic symptoms in their previous psychiatric episode, while the remaining 7 did not manifest these symptoms.

**Table 1:** Description of the groups of patients and controls.

		Patients	Controls
Age (mean ± SD), years		35.5±9.6	34.9±10.1
Women		N = 22	N = 18
Men		N = 15	N = 12
With psychotic symptoms		N = 30	
COMT genotype	Val/Val	N = 10	
	Val/Met	N = 20	
	Met/Met	N = 6	

N: number of individuals.

One DNA sample was lost.

Table 2 shows and compares the TCI scores of bipolar patients and controls. We found that even though patients were euthymic, they scored lower in the dimensions of persistence, self-directedness and cooperativeness and higher in the harm avoidance and self-transcendence dimensions compared to controls.

**Table 2:** TCI scores (mean ± sd) of patients and controls.

Personality Dimensions		Patients N = 37	Controls N = 30	t	p	
Temperament	Novelty Seeking	56.2±10.2	58.0±8.6	0.78	0.43	
Dimensions	Harm Avoidance	61.3±12.5	55.3±8.1	2.25	0.027	*
	Reward Dependence	71.7±9.8	73.9±5.9	1.10	0.27	
	Persistence	60.4±15.1	67.1±8.9	2.15	0.03	*
Character	Self-Directedness	68.8±13.3	79.3±8.1	3.79	<0.001	*
Dimensions	Cooperativeness	77.1±9.1	83.9±6.5	W = 798	0.002	*
	Self-Transcendence	45.3±12.8	32.7±8.2	4.64	<0.001	*

N = number of individuals.

t = Student’s t test.

W = Mann-Whitney test.

* denotes a significant difference between patients and controls.

Table 3 shows the TCI dimensions in which there were statistically significant effects of COMT genotype. The COMT genotype significantly influenced the items novelty seeking, self-directedness, and cooperativeness. The values in the novelty seeking dimension were greater in the Met/Met genotype than in the Val/Val genotype. In the self-directedness dimension, the score was lower in the Met/Met genotype than in the Val/Met genotype. Regarding the cooperativeness dimension, patients with the Val/Val genotype showed lower scores than those with the Val/Met genotype. There was also an almost significant tendency of gender (p = 0.055) and age (p = 0.063) to influence novelty seeking values. These values were lower in women than in men and lower in older individuals.

**Table 3:** Multivariate analysis of the influence of the COMT genotype on TCI traits in bipolar patients.

Personality Dimension	COMT genotype						Model	
	Val/Val		Val/Met		Met/Met			
	mean	95% CI	mean	95% CI	mean	95% CI	F	p
NS	50.0	44.2–55.8	56.5	52.4–60.6	66.3	58.8–73.6	6.18	0.0052
SD	64.6	56.7–72.5	73.8	68.2–79.4	58.2	47.9–68.4	4.49	0.0189
C	70.7	65.3–76.0	80.1	76.4–83.9	78.8	71.9–85.7	4.44	0.0196

The table shows the mean values and the 95% confidence intervals for the TCI dimensions in which there were statistically significant effects of COMT genotype.

NS: Novelty seeking. The values of the Met/Met genotype are greater than the Val/Val genotype.

SD: Self-Directedness. The values of the Met/Met genotype are lower than the Val/Met genotype.

C: Cooperativeness. The values of the Val/Val genotype are lower than the Val/Met genotype.

## Discussion

### 1. Patient and Control Group Comparison

The high values in harm avoidance observed in our study in bipolar patients with respect to healthy controls are consistent with findings in numerous studies with different types of bipolar patients: euthymic patients and patients that remain symptomatic or are in the process of recovery [2], [3], [5], [6], [7], [22], [23], [24], [25], [26], [27]. Although some authors have not found this higher value in the harm avoidance dimension of euthymic bipolar patients [4], [28], other authors have observed this temperamental characteristic even when using different versions of the TCI. However, it cannot be concluded that this is syndrome-specific because high scores in this temperamental dimension have also been observed, for example, in schizophrenic patients [29], patients with major depression [27] and in other psychiatric disorders [30]. Thus, it is possible that a greater value in harm avoidance is a genetic marker of a vulnerability to suffer from a psychiatric disorder, but not BD in particular.

Another temperamental trait that differed between the patient and control group was persistence. In our study, persistence shows a lower value in BD patients. In some other studies, no significant differences are found when comparing the patient and control group [2], [5], [7], [22], [24], [25], [27], [28], although some findings do coincide with ours [3], [4], [6]. Differences in methodologies can explain these discrepancies, such as the type of interview that was used to evaluate personality. Some studies [5], [22] use the Tridimensional Personality Questionnaire, in which the persistence trait is included in the dimension of reward dependence. Other differences in the results obtained might derive from the psychopathological state the patient is in when the questionnaire is applied, the diagnosis of BD, or the episode within the diagnosis of BD. The studies that detect a lower value in persistence all apply to euthymic patients [3], [4], [6]. Persevering and goal-directed behaviors characteristic of high persistence have been suggested to increase when patients are in a manic or hypomanic state [7]. Notably, in other studies with euthymic patients, this difference has not been reported [2], [27], [28]. Thus, as occurred with the harm avoidance dimension, it is possible that a low value in persistence is a marker of a certain subgroup of bipolar patients or affective disorders because lower values in persistence have been found in patients with major depression [31]. In most of the studies with schizophrenic patients, this value does not decrease [32], and when it does, it appears to be associated with the presence of negative symptomatology [33], [34].

Regarding the character dimensions, the patients in our study show a lower score in self-directedness and cooperativeness and a greater score in self-transcendence when compared to the controls. These results agree with various studies on bipolar patients [6], [26], [35]. Higher values in self-transcendence and lower values in self-directedness have been associated with the presence of psychotic symptoms [7], [33], [36] or the antipsychotic dosage [32]. It is possible that the antipsychotic treatment or presence of psychotic symptoms in most of our patients could explain the higher self-transcendence scores or the lower self-directedness scores. However, the limited number of patients in our sample without psychotic symptoms during their previous episode precludes us from evaluating the possible influence of psychotic symptoms on those character dimensions. However, these same differences in character traits have been observed in depressive patients [31], [37], [38]. Therefore, it seems that suffering from an affective disorder could have an impact on the character of these patients.

Lithium may influence the patterns of personality in bipolar patients. However, several studies [3], [4], [5] of bipolar patients with and without lithium treatment have found similar results in personality. It has also been reported that effective antidepressant treatment influences personality dimensions [31] but our patients were not receiving antidepressants. Thus, it seems likely that the differences reported between bipolar patients and healthy controls cannot be explained by an effect of the mood stabilizer treatment.

In Table 4, we present a summary of the TCI findings in BD and healthy controls collected from the references cited in this section. We present only the TCI traits that we found to be different between patients and controls. Most of these studies were performed in larger samples than our own; however, some included both bipolar I and II patients, and in some, the patients were not all euthymic. Furthermore, some of these studies simultaneously examined additional diagnostic groups. Our sample, although small, was homogenous in terms of clinical state and diagnosis. It may represent a subgroup of bipolar patients similar to a subset of patients described in larger studies.

**Table 4:** Summary of the TCI findings in BD and healthy controls from previous studies. We present only the TCI traits that we have found to be significantly different in bipolar patients and healthy controls.

	Patients				Healthy Controls		Comparison of patients versus healthy controls					
Reference	Diagnostic	N	Euthymic		N	Formally evaluated	HA	P	SD	C	S	
			Yes	Not								
2	BP I	75	X		100		BP I n.s.	BP I n.s.	BP I n.s.	BP I **−**	BP I n.s.	
	BP II	25					BP **+**.	BP n.s.	BP **−**	BP **−**	BP n.s.	
3	BP	50	X		1019		BP **+**	BP **−**	n.m.	n.m.	n.m.	
4	BP	25	X		25		n.s.	BP **−**	n.m.	n.m.	n.m.	
5 *	BP	45	HRSD<10		100		BP **+**	n.m.	n.m.	n.m.	n.m.	
			YMRS<10									
6	BP I	99	X	X	264		BP **+**	BP **−**	BP **−**	BP **−**	n.s.	
	BP II	92										
7	BP I	85	HRDS<17		85	Yes	BP I **+**	n.s.	BP I **−**	n.s.	BP I **+**	
			MADRS<15									
22	BP	40	X		89		BP **+**	n.m.	n.m.	n.m.	n.m.	
23 *	BP I	109	X	X	63		BP **+**	n.s.	BP **−**	BP **−**	BP **+**	
	BP II	46										
24 *	BP I	50	X	X	87(UR)	Yes	BP I **+**	n.s.	BP I **−**	n.s.	BP I **+**	
	BP II	15					BP II **+**	n.s.	BP II **−**	n.s.	n.s.	
25 *	BP II	21	X	X	21		BP II **+**	n.s.	BP II **−**	n.s.	n.s.	
26	BP	73	X	X	63	Yes	BP **+**	n.s.	BP **−**	BP **−**	BP **+**	
27 *	BP	49	X		47	Yes	BP **+**	n.s.	BP **−**	n.s.	n.s.	
28	BP I	81	HRSD<7		90	Yes	n.s.	n.s.	BP **−**	BP **−**	n.s.	
	BP II	9	YMRS<13									
Present	BP I	37	HRSD<8		30		BP I +	BP I **−**	BP I **−**	BP I **−**	BP I +	
study			YMRS<10									

N: number of individuals.

HA: Harm Avoidance. P: Persistence. SD: Self-Directedness. C: Cooperativeness. ST: Self-Transcendence.

BP I: bipolar I patients. BP II: bipolar II patients. BP: BP I+BP II.

**+** Higher in patients than in controls.

**−** Lower in patients than in controls.

n.s. non significant difference between patients and controls. n.m. not measured.

* Other diagnostic groups were studied in addition to bipolar patients.

HRSD Hamilton’s Rating Scale for Depression.

MADRS Montgomery-Åsberg Depression Rating Scale.

YMRS Young’s Mania Rating Scale.

UR = unaffected relatives.

### 2. The Relationship between Genotype and Personality

The novelty seeking temperament dimension has been associated with the COMT Val158Met genotype. Patients with the low catabolic activity genotype, Met/Met, have a higher score in novelty seeking than patient carriers of the high catabolic activity genotype, Val/Val. To the best of our knowledge, this is the first study with bipolar patients in which this association has been detected. The results are consistent with the findings of other studies with healthy controls of Russian [39] or Hungarian origin [40] and with findings in meta-amphetamine-dependent [41]or heroin-dependent Europeans [40]. Other studies have not detected this association [20], [21], [42], [43], [44] or even detected an association between the Val/Val genotype and higher novelty seeking scores [45]. However, most of these studies have been performed with the Asian population, in which the proportion of Met alleles is lower to that of other population groups [46], which could explain the existing discrepancies between the studies. In studies that have been performed in depressive patients, mainly of European descent, this association has not been found [47].

The results of higher novelty seeking scores in individuals with low activity alleles in the COMT gene are compatible with Cloninger’s proposal. The authors [48] link the novelty seeking dimension with the dopaminergic system and the role of dopamine in the brain’s reward circuits. Dopamine released in the nucleus accumbens is thought to contribute to the decision to exert effort to seek reward. COMT’s involvement in the inactivation of dopamine is of greater relevance in the prefrontal cortex where no dopamine transporter exists. A low activity of COMT in this zone has been suggested to entail a greater frontal dopaminergic activity and a lower dopaminergic activity in the mesolimbic dopaminergic system [49]. In turn, this would translate into a greater need to seek novelties that would raise dopamine to optimum levels. Alternatively, the effect could also be due to another polymorphism of functional significance in linkage disequilibrium with Val158Met.

The COMT enzyme also participates in the inactivation of noradrenaline, and this neurotransmitter has been associated with the dimension of reward dependence. In our study, we have found no relationship between this dimension and the Val158Met genotype. This finding could be because this enzyme is of less importance in the inactivation of noradrenaline due to monoamine oxidase playing a large role in the inactivation of noradrenaline [50].

The relationship between character traits and the Val158Met COMT genotype is more difficult to interpret because there is an association between the low activity genotype, Met/Met, and a lower self-directedness and the high activity genotype, Val/Val, and a lower cooperativeness when compared to the intermediate activity genotype Val/Met. An inverted U-shaped association between the dopaminergic activity associated with the Val158Met genotype and its related function has been observed [51]. Recently, an inverted U-shaped correlation between sensation seeking, a personality trait, and striatal dopamine receptor availability has been reported [52]. Although highly speculative, it is possible that an inverted U-shaped relation exists between self-directedness and cooperativeness scores and the dopaminergic activity associated with COMT genotypes. The intermediate activity COMT genotype (Val/Met) would be at the top of this curve. We also speculate that if character is the result of genetic and environmental factors, the influence of life events on personality may be modulated by COMT genotype. The COMT Val158Met genotype has been found to influence the development of adult psychosis after adolescent cannabis use [53] and to affect paranoid reactivity to minor stressors in daily life [54]. Alternatively, this relationship could be an artifact, or the size of our sample could be insufficient and does not allow us to detect differences between the genotypes.

There are fewer studies on the relationship between genotypes and character traits than on genotypes and temperament. In women with eating disorders, the Val/Val genotype has been associated with a lower cooperativeness and self-directedness [55]. If character traits vary progressively depending on individual life experiences, the association found could be due to the role played by the Val158Met genotype on the expression of BD or on the different brain functions. For example, the Met/Met genotype has been associated with better cognitive abilities [56], and the Val/Val has been associated with a greater presence of psychotic symptomatology in BD patients [12], [13].

### 3. Limitations

The size of our study is small but similar to those of other studies that have detected associations between personality and BD [4], [25] or between personality and genotype [41], [57]. Table 4 shows a comparison of several studies that have reported differences in personality between bipolar patients and healthy controls; our sample is smaller than most of these studies but has the advantage of being homogenous in terms of diagnosis and clinical state. In contrast, our study may be based on a subgroup of bipolar patients that may not be generalizable to the bipolar disorder population.

Another limitation is that we did not evaluate the COMT genotypes of the healthy control group. Regardless, an association between COMT genotype and novelty seeking has been detected in other studies of healthy controls of the same ethnic origin as our sample [40]. The healthy control participants were not evaluated following a formal diagnostic test; however, they were recruited from the hospital staff and medical students and interviewed by a specialist.

A complex interaction between the variable number of repeats in the D4 dopamine receptor exon III, COMT Val158Met, serotonin transporter promoter length polymorphisms and novelty seeking has been reported [58], [59]. We could not examine this interaction due to our sample size.

### 4. Conclusion

In summary, we show that bipolar patients scored higher in harm avoidance and self-transcendence and lower in persistence, self-directedness and cooperativeness compared to controls. Greater harm avoidance scores may be a marker of vulnerability to some psychiatric disorders but not for BD in particular, and a low score in persistence may be associated with a subgroup of patients with BD or affective disorders. Regarding character traits, higher self-transcendence and lower self-directedness scores have also been detected in studies of depressive patients, and this finding is compatible with the idea that affective disorders affect the character of these patients. It is also possible that the presence of psychotic symptoms in most of our patients could explain the differences in these character traits.

Bipolar patients with the low catabolic activity COMT genotype (Met/Met) have higher novelty seeking scores than patient carriers of the high catabolic activity genotype (Val/Val) This result is consistent with findings in healthy controls and drug abusers of European descent. It is also consistent with the proposed link between novelty seeking and the dopaminergic system. Regarding character traits, when compared to the intermediate activity genotype (Val/Met), we found associations between the Met/Met genotype and lower self-directedness and the Val/Val genotype and lower cooperativeness.
